# HPV-related anal cancer is associated with changes in the anorectal microbiome during cancer development

**DOI:** 10.3389/fimmu.2023.1051431

**Published:** 2023-03-29

**Authors:** Jacob H. Elnaggar, Victoria O. Huynh, Daniel Lin, R. Tyler Hillman, Chike O. Abana, Molly B. El Alam, Katarina C. Tomasic, Tatiana V. Karpinets, Ramez Kouzy, Jae L. Phan, Jennifer Wargo, Emma B. Holliday, Prajnan Das, Melissa P. Mezzari, Nadim J. Ajami, Erica J. Lynn, Bruce D. Minsky, Van K. Morris, Andrea Milbourne, Craig A. Messick, Ann H. Klopp, P. Andrew Futreal, Cullen M. Taniguchi, Kathleen M. Schmeler, Lauren E. Colbert

**Affiliations:** ^1^ School of Medicine, Louisiana State University Health Sciences Center, New Orleans, LA, United States; ^2^ Department of Radiation Oncology, The University of Texas MD Anderson Cancer Center, Houston, TX, United States; ^3^ Department of Gynecologic Oncology and Reproductive Medicine, The University of Texas MD Anderson Cancer Center, Houston, TX, United States; ^4^ Department of Genomic Medicine, The University of Texas MD Anderson Cancer Center, Houston, TX, United States; ^5^ Cancer Prevention Research Institute of Texas Scholar in Cancer Research, Austin, TX, United States; ^6^ Department of Surgical Oncology, The University of Texas MD Anderson Cancer Center, Houston, TX, United States; ^7^ Gastrointestinal Radiation Oncology, The University of Texas MD Anderson Cancer Center, Houston, TX, United States; ^8^ The Alkek Center for Metagenomics and Microbiome Research, Department of Molecular Virology and Microbiology, Baylor College of Medicine, Houston, TX, United States; ^9^ Department of Gastrointestinal Medical Oncology, The University of Texas MD Anderson Cancer Center, Houston, TX, United States

**Keywords:** anal cancer, anorectal microbiome, HPV-related cancer, anal dysplasia, cancer biology

## Abstract

**Background:**

Squamous cell carcinoma of the anus (SCCA) is a rare gastrointestinal cancer. Factors associated with progression of HPV infection to anal dysplasia and cancer are unclear and screening guidelines and approaches for anal dysplasia are less clear than for cervical dysplasia. One potential contributing factor is the anorectal microbiome. In this study, we aimed to identify differences in anal microbiome composition in the settings of HPV infection, anal dysplasia, and anal cancer in this rare disease.

**Methods:**

Patients were enrolled in two prospective studies. Patients with anal dysplasia were part of a cross-sectional cohort that enrolled women with high-grade lower genital tract dysplasia. Anorectal tumor swabs were prospectively collected from patients with biopsy-confirmed locally advanced SCCA prior to receiving standard-of-care chemoradiotherapy (CRT). Patients with high-grade lower genital tract dysplasia without anal dysplasia were considered high-risk (HR Normal). 16S V4 rRNA Microbiome sequencing was performed for anal swabs. Alpha and Beta Diversity and composition were compared for HR Normal, anal dysplasia, and anal cancer.

**Results:**

60 patients with high-grade lower genital tract dysplasia were initially enrolled. Seven patients had concurrent anal dysplasia and 44 patients were considered HR Normal. Anorectal swabs from 21 patients with localized SCCA were included, sequenced, and analyzed in the study. Analysis of weighted and unweighted UniFrac distances demonstrated significant differences in microbial community composition between anal cancer and HR normal (p*=*0.018). LEfSe identified that all three groups exhibited differential enrichment of specific taxa. *Peptoniphilus* (p=0.028), *Fusobacteria* (p=0.0295)*, Porphyromonas* (p=0.034)*, and Prevotella* (p=0.029) were enriched in anal cancer specimens when compared to HR normal.

**Conclusion:**

Although alpha diversity was similar between HR Normal, dysplasia and cancer patients, composition differed significantly between the three groups. Increased anorectal *Peptoniphilus, Fusobacteria*, *Porphyromonas*, and *Prevotella* abundance were associated with anal cancer. These organisms have been reported in various gastrointestinal cancers with roles in facilitating the proinflammatory microenvironment and neoplasia progression. Future work should investigate a potential role of microbiome analysis in screening for anal dysplasia and investigation into potential mechanisms of how these microbial imbalances influence the immune system and anal carcinogenesis.

## 1 Introduction

Squamous cell carcinoma of the anus (SCCA) is a rare gastrointestinal cancer, affecting 8,000-9,000 patients annually in the US (1). It is linked to prior human papillomavirus (HPV) infections in approximately 90% of cases (2), but factors associated with the cancer development, such as the progression of HPV infection to dysplasia and anal cancer, are unclear. The standard-of-care treatment for patients with localized SCCA is chemoradiotherapy (CRT), which leads to 5-year survival rates of 75-90% (3). However, 30-40% of patients with advanced SCCA experience local recurrence or treatment-related toxicity (4, 5). Despite the use of HPV vaccines, the incidence and morbidity of SCCA continue to rise (6). Anal dysplasia is poorly understood and has been more difficult to detect than lower genital tract dysplasia. Women with HPV-related high-grade dysplasia or cancer of the cervix, vagina, or vulva are at increased risk for the development of anal dysplasia and SCCA (7–9). Prior observational studies have identified anal dysplasia in fewer than 15% of such high-risk women when assessed by high-resolution anoscopy (HRA), which is consistent with epidemiologic data showing that the incidence of anal cancer among women in the United States is much lower than the combined incidence of cervical, vaginal, or vulvar cancer (7, 10). Although some risk factors, such as tobacco use, are shared between both HPV-related diseases, the rarity of anal dysplasia and cancer suggests that additional external factors may modulate this risk. The identification of correlates between host genetics, environmental exposures, and the risk of anal dysplasia or cancer among high-risk patients would have important implications for cancer prevention efforts.

One potential contributing factor is the anal microbiome, which has been implicated in the progression of HPV infection to cancer in other HPV-related carcinogenesis (11). Knowledge about the pervasive influence of commensal bacteria led to the hypothesis that bacterial species found in the cervix and vagina contribute to the development of lower genital tract HPV infection, dysplasia, and cancer (11). Subsequent work using bacterial metagenomic sequencing methods has confirmed these observations by demonstrating a correlation between HPV-related cervical disease and lower levels of *Lactobacillus* species along with an increased abundance of anaerobic bacteria such as *Gardnerella vaginalis, Finegoldia magna*, *Atopobium vaginae, Dialister invisus, Prevotella buccalis, P. timonensis*, and *Fusobacterium* (12–15). Although the link between the microbiota and cervical carcinogenesis is well established, it is not yet known whether commensal bacteria play a role in the development of HPV-related disease at the anus.

Our previous work demonstrated a role for the anal microbiome in anal cancer response to therapy and toxicity (5). The ability to study the anal microbiome and carcinogenesis is limited by the rarity of the disease and limited patient samples. In this study, we combine anal microbiome samples collected from two prospectively enrolled cohorts, providing a unique opportunity to address this issue. The purpose of this study was to identify differences in anal microbiome composition in the settings of known HPV infection, anal dysplasia, and anal cancer.

## 2 Materials and methods

### 2.1 Study population and clinical assessments

**Figure 1:**
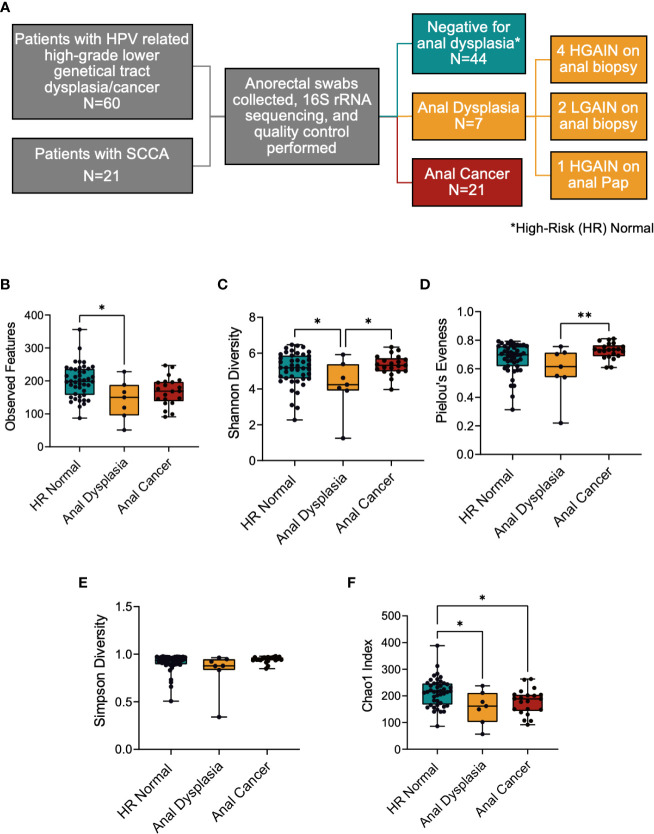
**(A)** Study overview depicting cohorts used and sample sizes. **(B–F)** Alpha diversity metrics between the three groups. **(B)** Richness of species was significantly decreased between HR Normal and Anal Dysplasia groups, **(C)** Shannon diversity richness index was lower in Anal Dysplasia, **(D)** Pielou’s Evenness index and **(E)** Simpson diversity index was greater in Anal Cancer compared to Anal Dysplasia, and **(F)** Chao1 was greater in HR Normal compared to both Anal Dysplasia and Anal Cancer. Statistical tests were performed using one way ANOVA. *P < 0.05, **P < 0.01. SCCA, Squamous cell carcinoma of the anus; HR Normal, High Risk Normal; H/LGAIN, high-grade or low-grade anal intraepithelial neoplasia.

We performed a retrospective analysis of anorectal microbiome composition using anorectal swab samples obtained from two prospective studies (**Figure 1A**). In the first study, we obtained anorectal swab samples from study participants enrolled between September 2016 and January 2017 in a cross-sectional study examining the prevalence of anal dysplasia among women with cervical, vaginal, and vulvar dysplasia at the University of Texas MD Anderson Cancer Center and Lyndon B. Johnson General Hospital (NCT02140021). Female patients were eligible for enrollment if they were over the age of 18 and had histologically confirmed cervical, vaginal, or vulvar high-grade dysplasia or invasive squamous cell carcinoma, invasive adenocarcinoma, or adenocarcinoma-*in-situ*. Pregnant women were excluded, as were any patients with previously documented perianal squamous cell dysplasia or invasive squamous cell carcinoma of the anus. We performed rigorous screening in order to minimize potential GI associated diseases that could alter the microbiome, such as excluding patients with previous abdominal or pelvic radiation therapy. This study was approved by the institutional review board at both the University of Texas MD Anderson Cancer Center (protocol PA2014-0021) and Lyndon B. Johnson General Hospital (eProtocol #14-05-0822).

In the second study, anorectal tumor swabs were prospectively collected from patients with biopsy-confirmed nonmetastatic SCCA receiving standard-of-care treatment as part of an institutional review board–approved study (protocol #2014-0543) at the University of Texas M.D. Anderson Cancer Center from April 2017 to July 2019 as described previously (5, 16). Patients with a previous history of abdominal or pelvic radiation were excluded from the study. Anal Pap smear and high resolution anoscopy were performed on all participants. Anorectal microbial specimens were collected in both studies using a swab biopsy technique.

Following the provision of written, informed consent, clinical and demographic data were collected at enrollment including age, menopausal status, self-reported ethnicity, HPV vaccination status, and human immunodeficiency virus (HIV) status. Information about prior diagnoses of cervical, vaginal, and vulvar dysplasia or cancer was also collected, along with the results of previous cervical or vaginal cytology and HPV testing. Information regarding the use of concurrent systemic antibiotics at the time of enrollment and tobacco use was obtained from the medical record.

Upon enrollment, in addition to standard of care treatment for genital tract disease, all anal dysplasia participants had anal cytology and anal HPV test samples collected, as well as an anorectal swab (Isohelix) prior to treatment for microbiome analysis. High-resolution anoscopy (HRA) was then performed as part of the study protocol. Biopsies were collected of abnormal areas identified by HRA as per standard of care practices. Participants were referred to a colorectal surgeon for anal high-grade anal squamous intraepithelial lesion (HSIL) anal cytology test result (regardless of HRA findings) or if biopsy of abnormal areas revealed high-grade or low-grade anal intraepithelial neoplasia (HGAIN, LGAIN, or AIN2-3). For the present analysis, patients were considered to have “anal dysplasia” if they had either high-grade or low-grade anal intraepithelial neoplasia (AIN1-3) on anal biopsy, or if their anal cytology showed HSIL regardless of HRA findings. Based on the clinical assessments, anal swab specimens were grouped as High-Risk (HR) Normal, Anal Dysplasia, or Anal Cancer (**Figure 1A**).

### 2.2 Assessment of rectal microbiome using 16S ribosomal RNA V4 region sequencing

Isohelix DNA Swabs (Isohelix, SK-2S) were used to collect tissue and fecal material from anal dysplasia and SCCA patients prior to standard of care treatment. Swab specimens were stabilized using BuccalFix DNA Stabilization Solution (Isohelix, BFX-25) within 1 hour of collection and stored at –80°C until DNA isolation. Amplicon sequencing of the 4^th^ hypervariable (V4) region of the bacterial 16S ribosomal RNA (rRNA) gene was performed on anorectal swabs by the Alkek Center for Metagenomics and Microbiome Research at Baylor College of Medicine, as previously described (16, 17). Bacterial genomic DNA was extracted from anorectal swabs using the MoBIO PowerSoil Kit (QIAGEN, 12855-50). The 16S rRNA gene sequencing methods were adapted from the Human Microbiome Project and Earth Microbiome Project (18). The bacterial 16S ribosomal RNA V4 genomic region was PCR amplified and sequenced using 250 bp paired-end reads on a MiSeq sequencer (Illumina, San Diego, CA). The primers used for amplification (515F-806R) contain adapters for MiSeq sequencing and single-index molecular barcodes so that the PCR products may be pooled and sequenced directly.

### 2.3 Sequencing read processing and taxonomic assignment

Sequencing data were processed and analyzed using QIIME2 v2022.2 (19). A QIIME2 provenance diagram is shown in **Figure S1A**. Sequencing quality control, amplicon sequence variant counts for feature table construction, and representative sequences used for phylogeny tree construction and taxonomic classification were performed using denoising *via* DADA2 (20) with the pseudo-pooling parameter and identical trim and truncation parameters of 0 and 188, respectively, across all samples. The trim and truncation settings were chosen based on quality scores generated by the QIIME2 platform. A rarefaction sampling depth of 11500 sequences per sample was used when necessary for downstream comparative analysis (**Figure S1B**). Nonrarefied data tables were used for linear discriminant analysis effect size. A naive Bayes classifier trained on 515/806R V4 ribosomal RNA regions from the SILVA release 132 database was used to assign taxonomy (21). Feature tables constructed using amplicon sequence variant counts were used for downstream comparative diversity analysis. Phylogenetic reference tree construction was performed using a SILVA 128 SATé-enabled phylogenetic placement database (22).

### 2.4 Microbial diversity metrics and microbiome composition

We analyzed the HR Normal, Anal Dysplasia, and Anal Cancer sequencing data using several different alpha diversity metrics (23). Observed Features (richness) provides a count of all identified putative species. Pielou’s evenness index calculates the proportions of individual species in a sample population. The Shannon diversity index accounts for the richness and evenness of taxa within a sample. The Simpson’s diversity index measures the diversity of species in a community. Faith’s phylogenetic diversity (PD) accounts for the phylogenetic differences between species in a sample. The inverse Simpson diversity index measures the relative counts of species that make up the richness, and finally, the Chao1 index estimates richness emphasizing rare species (23).

We generated stacked bar plots of genus level relative counts in ATIMA v3.1.2 (developed by the Center for Metagenomics and Microbiome Research at the Baylor College of Medicine) to observe taxa distribution across groups. Samples in these plots were ordered by relative counts of a specific taxon, e.g., *Bacteroides*. We also conducted compositional analysis using unweighted and weighted UniFrac and Bray Curtis to generate coordinates for each sample. Principal Coordinates Analysis (PCoA) and biplots were created in ATIMA to visualize group coordinates. Permutational multivariate analysis of variance (PERMANOVA) was used to assess differences in mean and variance between groups. Permutational multivariate analysis of dispersion (PERMDISP) was used to assess differences in dispersion between groups. Linear Discriminant Effect Size (LEfSe) (24) was performed in Miniconda v4.12 (25) was used to identify abundance changes of specific taxa that were enriched between all three groups. The LDA score cutoff was set at 3.5, and the alpha value for the Kruskal-Wallis test among classes was set at 0.05. Cladograms of the LEfSe results were generated to visualize and inspect the nestedness of the differentially enriched taxonomic groupings. Additionally, LEfSe was used with similar parameters to assess pairwise differences between groups. As a confirmatory test, DESeq2 was performed using pairwise comparisons across the groups using the contrast parameter (26), and volcano plots were made using the EnhancedVolcano package (**Figure S8**) (27). Heatmaps were made using the LEfSe results and colored by the group in which they were enriched. Taxonomic counts were log normalized and plotted using ComplexHeatmap (28). Stacked bar plots based on relative counts were also generated in ATIMA based on overall abundance and the LEfSe results.

### 2.5 Statistical analysis

For clinical and demographic variables, statistical comparisons were performed in RStudio (v2022.7.0.548) (29) using R v4.2.1 (30). A t-test was used for continuous variables. A Chi-squared test was used to compare categorical variables between HR Normal and Anal Cancer and Wilcoxon signed rank test was used for comparisons between Anal Dysplasia due to its smaller sample size. To compare alpha diversity, statistical tests were performed using pairwise one-way ANOVA to compare between the three groups HR Normal, Anal Dysplasia, and Anal Cancer; adjusted P<0.05 was considered statistically significant. Comparisons between differential abundance of specific taxa was performed on specific taxa using relative counts and compared using a similar one-way ANOVA. GraphPad Prism (GraphPad Software, v9.0) was used to generate graphs for data visualization.

## 3 Results

### 3.1 Patient characteristics

60 patients with high-grade lower genital tract dysplasia were initially enrolled. After excluding patients taking concurrent antibiotics and applying quality filters, anorectal swabs from 51 patients with high-grade lower genital tract dysplasia were sequenced. Of those, 7 patients had concurrent anal dysplasia, 4 HGAIN and 2 LGAIN on anal biopsy and 1 HGAIN on anal Pap. Additionally, this study included anorectal swabs from 21 patients with localized SCCA. In total, 71 swabs (44 HR Normal, 7 Anal Dysplasia, and 21 Anal Cancer) were included, sequenced, and analyzed in the study (**Figure 1A**). Since this study combines two cohorts and three stages of disease, there are noteworthy differences in patient demographics that are outlined in **Table 1**. These are grouped by sample type. The age in the HR Normal group (mean=46.1 years) was statically lower than the Anal Cancer group (mean=57 years, P<0.001). However, there was no difference between the Anal Dysplasia group (mean=54 years). Of the participants in the Anal Cancer group, 3 (16.67%) were males. The rest of the participants in the study were females. Additionally, there were differences in other categories including site of recruitment, menopausal status, and ethnicity. Also, one Anal Cancer patient reported a positive HIV diagnosis. Additional clinical and demographic information can be found in **Table S1**.

**Table 1:** Patient Characteristics.

	HR Normal	Anal Dysplasia	Anal Cancer	P value
	N=44	N=7	N=21	
**Age**				< 0.001^a^
mean	46.09^a^	53.71	57.38^a^	
median	44.5	58	60	
min	24	32	39	
max	72	67	77	
**Gender**				
F	44^b^	7	18^b^	
M	0	0	3	
**Race**				
White	40	5	16	
Black	3	1	3	
Asian	1	1	1	
Other	0	0	1	
**Menopausal Status**				0.010^b^
Post-Menopausal	22^b^	5	16^b^	
Pre-Menopausal	22	2	2	
NA	0	0	3	
**Ethnicity**				0.016^b^
Hispanic or Latino	16^b^	1	1^b^	
Not Hispanic or Latino	28	6	20	
**Study Center**				0.023^b^
LBJ	12^b^	1	0^b^	
MDACC	32	6	21	
**Current Tobacco Use**				
No	40	6	21	
Yes	4	1	0	
**Current ABX**				
No	39^b^	6	20^b^	
Yes	5	1	0	
Missing	0	0	1	
**HIV Status**				
Positive	0	0	1	
Negative	43	7	16	
Missing	1	0	4	

P value determined by (a) test or (b) Chi-squared test. All statistical tests were two-sided. HR Normal, High-Risk Normal; F, female; M, male; LBJ, Lyndon B. Johnson; MDACC, MD Anderson Cancer Center; ABX, antibiotic usage.

### 3.2 Alpha diversity

In general, the Alpha diversity metrics (**Figures 1B–F**) displayed a similar pattern of changes among the 3 groups, namely, the greatest was HR Normal, the lowest being Anal Dysplasia, and the intermediate value was Anal Cancer. Specific comparisons revealed that the number of observed features was increased in the HR Normal (mean=119.2) specimens when compared to Anal Dysplasia (mean=142.4, P=0.017, **Figure 1B**). The Shannon diversity index for Anal Dysplasia (mean=4.169) was significantly lower when compared to both HR Normal (mean= 5.11, P=0.042) and Anal Cancer specimens (mean=5.34, P=0.015, **Figure 1C**). Additionally, Anal Dysplasia was lower than Anal Cancer for Pielou’s Evenness (Anal Dysplasia mean=0.585, Anal Cancer mean= 0.7255 P=0.019, **Figure 1D**) and Simpson Diversity indexes (Anal Dysplasia mean=0.8224; Anal Cancer mean=0.9427, P=0.019, **Figure 1E**). By Chao1 (**Figure 1F**), HR Normal (mean=213.7) was significantly higher than both Anal Dysplasia (mean=156.7, P=0.026) and Anal Cancer (mean=177.8, P=0.032). Other metrics include Faith PD and Inverse Simpson (**Figure S2**), and all per sample diversity values and P values can be found in **Table S1**.

### 3.3 Beta diversity

**Figure 2:**
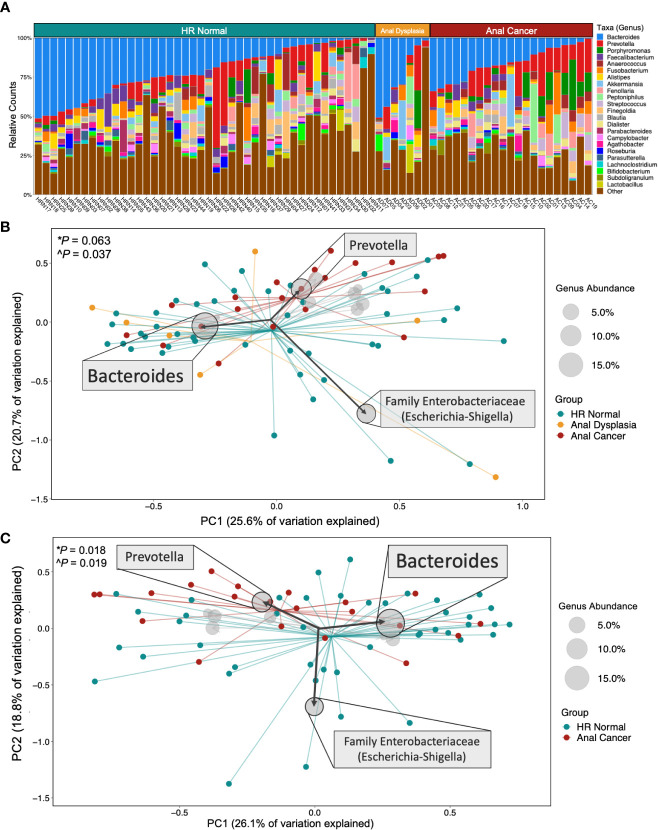
Microbial composition and beta diversity across groups. **(A)** Stacked bar plot of relative counts for all samples in the study. **(B)** Comparing all three groups and **(C)** HR Normal vs Anal Cancer by Weighted UniFrac Principal Coordinate Analysis and biplot showing taxa that pulled samples in a specific direction. Centroids display converging points for each group. The size of the circle represents the abundance of that taxa across all samples displayed, and specific taxa were displayed based on abundance. Statistical tests used *PERMANOVA and *^PERMDISP.* HR Normal, High Risk Normal; PC, principal component.

There is a large proportion of the genus *Bacteroides* among all three groups (blue, **Figures 2A**, **S3A**), as well as *Prevotella* (red, **Figure S3B**) and the class Clostridia (orange, **Figure S3G**). Analysis of weighted UniFrac distances demonstrated a significant dispersion in microbial community composition between the three groups (**Figure 2B**, PERMANOVA P*=*0.063, PERMDISP P=0.037). Unweighted UniFrac and weighted Bray Curtis comparisons revealed significant differences amongst all three groups (PERMANOVA P*=*0.001, P=0.020; PERMDISP P=0.557, P=0.902; **Figures S4B, C**). There was also a significant difference between HR Normal and Anal Cancer (**Figure 2C** PERMANOVA P*=*0.018, PERMDISP P=0.019). All PCoA metric comparisons can be observed in **Table S1** with corresponding figures in **Figure S4**. A weighted UniFrac biplot of the three group comparisons (**Figure 2B**) displayed a pull from the family *Enterobacteriaceae* (comprising the genera Escherichia and Shigella). There was also a split in the larger cluster between *Bacteroides* and *Prevotella*. These findings are also replicated in the pairwise biplot between HR Normal and Anal Cancer (**Figure 2C**). Additional comparisons using additional metrics and alternative taxonomic levels can be observed in **Figure S4**.

### 3.4 Microbial composition and abundances

**Figure 3:**
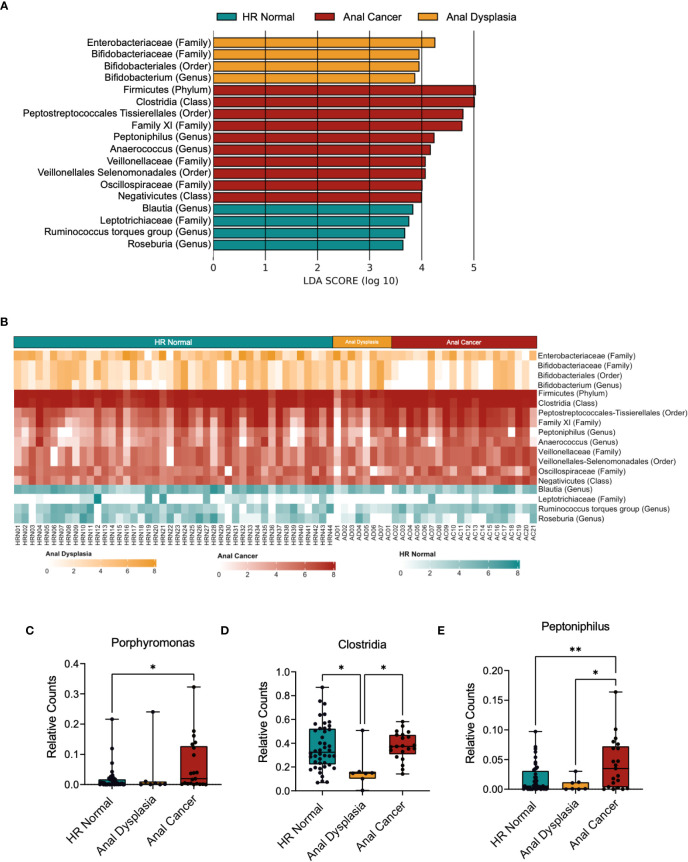
Taxa-specific changes across three groups. **(A)** Linear discriminant analysis (LDA) effect size (LEfSe) derived bar graph of enriched taxa between HR Normal, Anal Dysplasia, and Anal Cancer. An LDA of 3.5 or greater was used as a cutoff and an alpha of 0.05 for the Wilcoxon rank-sum test. **(B)** Heatmap colored horizontally based on the group each taxon was enriched from LEfSe. Values were log normalized. **(C–E)** Comparison of relative counts between the three groups for specific taxa. Statistical tests were performed using one way ANOVA. *P < 0.05. **P < 0.01. Abv. HR Normal: High Risk Normal.

**Figure 4:**
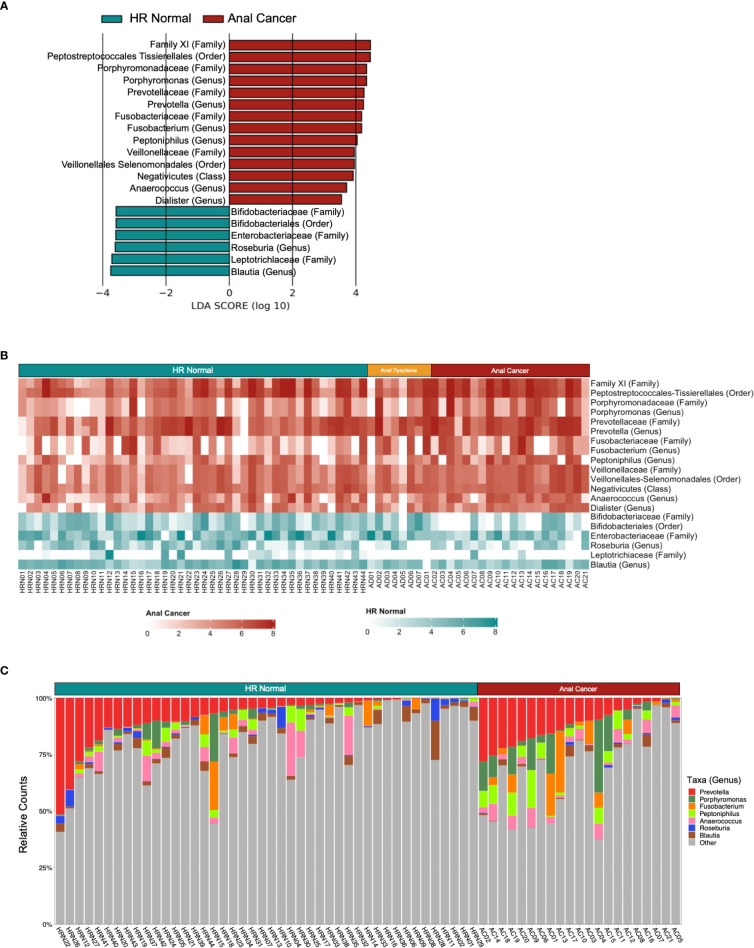
Differently abundant taxa between HR Normal and Anal Cancer. **(A)** Linear discriminant analysis (LDA) effect size (LEfSe) derived bar graph of enriched taxa between HR Normal and Anal Cancer. **(B)** Heatmap colored horizontally based on the group each taxon was enriched from LEfSe. Values were log normalized. **(C)** Stacked bar plot of relative counts for HR Normal and Anal Cancer highlighting the genera enriched from LEfSe. An LDA of 3.5 or greater was used as a cutoff and an alpha of 0.05 for the Wilcoxon rank-sum test.

LEfSe identified that all three groups exhibited differential enrichment of specific taxa (**Figure 3A**, **S5A**; **Table S1**), including increased abundance of *Peptoniphilus* (p=0.025), Firmicutes (p=0.037345458), *Anaerococcus* (p=0.016), *Oscillospiraceae* (p=0.043) and Clostridia (p=0.013) among Anal Cancer, Bifidobacterium (p=0.048) among Anal Dysplasia, and *Roseburia* (p=0.035) and *Blautia* (p=0.021) among HR Normal. Pairwise comparison between Anal Cancer and HR Normal (**Figures 4A, B**) showed a similar increased abundance of Peptoniphilus (p=0.025) and Anaerococcus (p=0.023) and additional organisms including *Porphyromonas* (p=0.027), *Prevotella* (p=0.023), and *Fusobacterium* (p=0.028). Anal Cancer also displayed an increased abundance of Clostridia, Firmicutes, *Peptoniphilus*, Anaerococcus, and *Oscillospiraceae* when compared to Anal Dysplasia (**Figure S5**).

Using the LEfSe to guide further analyses, we compared taxonomic abundances between the three groups. Heatmaps can be observed comparing the three groups and (**Figure 3B**) and comparing HR Normal to Anal Cancer (**Figure 4B**). The groups are colored horizontally based on the group in which they were found to be enriched from LEfSe (**Figures 3A**, **4A**). A stacked bar plot using the genus level enrichments from LEfSe can be observed in **Figure 4C**, and family level in **Figure S6**.

When comparing relative counts of specific organisms, there was a significant increase in *Porphyromonas* between HR Normal (mean=0.0169) and Anal Cancer (mean=0.0629, P=0.015, **Figure 3C**) which can also be visually observed (green, **Figure 4C**). There was also significant decrease in *Clostridia* between Anal Dysplasia (mean=0.1741) and both HR Normal (mean=0.3679, P=0.019, **Figure 3D**) and Anal Cancer (mean=0.3774, P=0.022). And finally, there was a significant increase in *Peptoniphilus* in Anal Cancer (mean=0.0423) compared to HR Normal (mean=0.0171, P=0.006) and Anal Dysplasia (mean=0.0077, P=0.027, **Figure 3E**). Similar comparisons were performed for *Anaerococcus, Fusobacterium, Lachnospiraceae, Oscillospirales, Oscillospiraceae*, and *Prevotella* but were not significantly different between groups (**Figure S7**; **Table S1**).

## 4 Discussion

Although the microbiome has been well characterized in some HPV-driven cancers, establishing an association between anal cancer and the anorectal microbiome remains difficult due to the rarity of disease and limited patient specimens. We observed primarily compositional and beta diversity differences between our HR Normal, Anal Dysplasia, and Anal Cancer samples with some differences in richness and evenness. We also noted significant enrichment of specific taxa among the Anal Cancer group in comparison to HR Normal, including *Peptoniphilus, Prevotella*, *Porphyromonas, and Fusobacterium* (**Figures 3**, **4**).

In patients with anal cancer, we observed an increase in the abundance of the genera *Peptoniphilus (*a member of the Clostridia class)*, Prevotella*, *Porphyromonas*, and *Fusobacterium*, which are all linked to a range of pro-inflammatory and immune modulating functions. *Peptoniphilus* is a gram-positive anaerobic commensal that colonizes mucosal surfaces, such as the mouth, gastrointestinal tract, and genitourinary tract. There are reports of a higher abundance of *Peptoniphilus* in colorectal cancer (CRC) (31), and it is a highly opportunistic pathogenic bacteria that produces calprotectin (32, 33), a protein specific to neutrophil mediated inflammation and neutrophil recruitment (34). *Prevotella* is an anaerobic gram-negative that inhabits the oral cavity, vagina, and gut and is associated with chronic inflammation (35–37). *Prevotella* abundance is associated with T helper type 17 (Th17) and T helper type 1 (Th1) mediated mucosal inflammation and immune response through activation of toll-like receptor 2 (38), in addition to stimulating epithelial cells to produce chemokines and cytokines (39, 40). In one study examining the microbiome of men who have sex with men (MSM) patients with anal precancerous lesions, *Prevotella* was one of the most predictive bacteria associated with high-grade squamous intraepithelial lesions (41). Another study showed an association of *Prevotella* abundance with persistent cervicovaginal HPV infection (42). *Porphyromonas* also plays a significant role in CRC (43–46) and is associated with a significant increase in TNF-alpha and IL-6 (47). More specifically, *Porphyromonas gingivalis* has been well-associated with the occurrence and development of gastrointestinal cancers. In mice orally administered *Porphyromonas gingivalis*, the mRNA levels of IFN-gamma were elevated in the gut (48). In CRC, *Fusobacterium* is associated with tumor carcinogenesis, high disease stage, poor tumor differentiation, poor prognosis (49) and metastatic disease (50, 51). Reported mechanisms for these associations in CRC include 1) TLR4-induced initiation of signaling pathways that lead to the secretion of inflammatory cytokines such as TNF-alpha and IL-8 (52, 53), 2) expression of NF-κB, which inhibits apoptosis and stimulates cell proliferation (54), 3) NK T-cell inhibition (55), and 4) recruitment of myeloid-derived suppressor cells that suppress CD4 T-cells (56, 57). We propose that *Fusobacterium*, along with these other taxa, could affect immune function in anal carcinogenesis and cancer.

This study has several strengths and limitations. A key strength is the inclusion of a relatively homogenous group of patients with a rare disease who share an important risk factor for anal dysplasia, specifically the co-existence of high-grade dysplasia of the lower genital tract. An additional strength of this study is the minimization of ascertainment bias attained through the performance of anal cytology tests and HPV testing as well as HRA on all study participants, consistent with the current diagnostic standard for the detection of anal dysplasia (58). Several limitations exist, however, primarily due to the small sample size given the rarity of anal cancer and anal dysplasia, and sequencing methodology. We can make stronger comparisons between High Risk Normal and Anal Cancer due to their larger sample sizes, while comparisons with Anal Dysplasia are more limited. Nevertheless, these findings are not conclusive. This study is aimed to serve as a pilot for future research in this area. Individual genus composition alone does not fully describe potential interactions between these bacterial genera that could drive the shift from HPV infection to dysplasia and cancer. Although evaluation of co-occurrences of these species in this study is limited by the small sample size, several of these genera are known to interact and function in synergy. For example, *Peptoniphilus* and *Prevotella* are found to co-occur in association with recurrence of bacterial vaginosis (29), elevated risk for HIV seroconversion and HIV antiviral treatment resistance (38). In addition, *Fusobacterium*, *Porphyromonas*, and *Prevotella* have been co-associated together in colorectal cancer (59, 60). Another remaining unknown from this study is a description of the function of the individual bacterial species associated with anal cancer. Performing a functional analysis would require shotgun metagenomic sequencing rather than 16S sequencing. However 16S sequencing is superior for compositional analysis (61), which was the primary goal of this study. Future work utilizing whole genome sequencing will describe the functional composition and metabolic analysis of this patient population.

There are significant differences in demographics between HR Normal and Anal Cancer (**Table 1**). Anal cancer tends to develop later than cervical cancer (62). Therefore, age, and as a result, menopausal status can differ. The Lyndon B. Johnson General Hospital generally cares for a more diverse population which may explain the difference in ethnicity between the two groups. Additionally, there was an Anal Cancer patient with HIV. There an increased risk of HPV-associated cancers, including anal cancer, among individuals with HIV as well as an increased risk with immunosuppression (63). Due to the rarity of anal dysplasia and cancer, a necessary limitation of this study is the small sample size and imbalance between the number of subjects in each group. However, the rate of anal dysplasia in this cohort is consistent with prior reports of incidence in high-risk women (7). In order to mitigate these limitations, we employed rigorous statistical procedures for FDR control and stringent cut-offs for multiple testing. Although this study can provide only exploratory suggestions of correlation due to its design, these results are important for hypothesis generation to guide further studies in this area. Additionally, we plan to perform functional analysis and experimental studies to test these specific hypotheses. Future prospective studies are needed to confirm the generalizability of the observed association between specific bacterial abundance and anal cancer development including mechanisms by which the anorectal microbiota may influence anal dysplasia and cancer risk.

Overall, our findings suggest an association between the anorectal microbiome among anal cancer development. Our work highlights potential roles in an understudied disease that needs to be further explored. Implications of this work could include improved diagnostic tools for this rare and difficult to detect disease, especially among high-risk patients. This could allow us to intervene early and prevent anal dysplasia and the progression to cancer.

## Data availability statement

The data presented in the study are deposited in the NCBI Sequence Read Archive repository, accession number PRJNA880301.

## Ethics statement

The studies involving human participants were reviewed and approved by institutional review boards at both the University of Texas MD Anderson Cancer Center (protocol PA2014-0021 & #2014-0543) and Lyndon B. Johnson General Hospital (eProtocol #14-05-0822). The patients/participants provided their written informed consent to participate in this study.

## Author contributions

RH, PF, KS, AK, CT, and LC conceived the study. JE, DL, RH, VH, TK, LC, and NA analyzed data. AM, CM, KS, AK, CT, and LC collected samples. AM, CM, AK, JW, PF, KS, and LC jointly supervised the study and provided oversight during the manuscript editing process. JE and VH wrote the first draft and revised the manuscript for submission. All authors contributed to the article and approved the submitted version.
