# Supplementary material for: HPV-related anal cancer is associated with changes in the anorectal microbiome during cancer development
Source: Front Immunol. 2023 Mar 29;14:1051431. doi: 10.3389/fimmu.2023.1051431 (PMC10090447; doi:10.3389/fimmu.2023.1051431)

# Supplemental Figure 1

**A** QIIME2 microbiome analysis provenance diagram

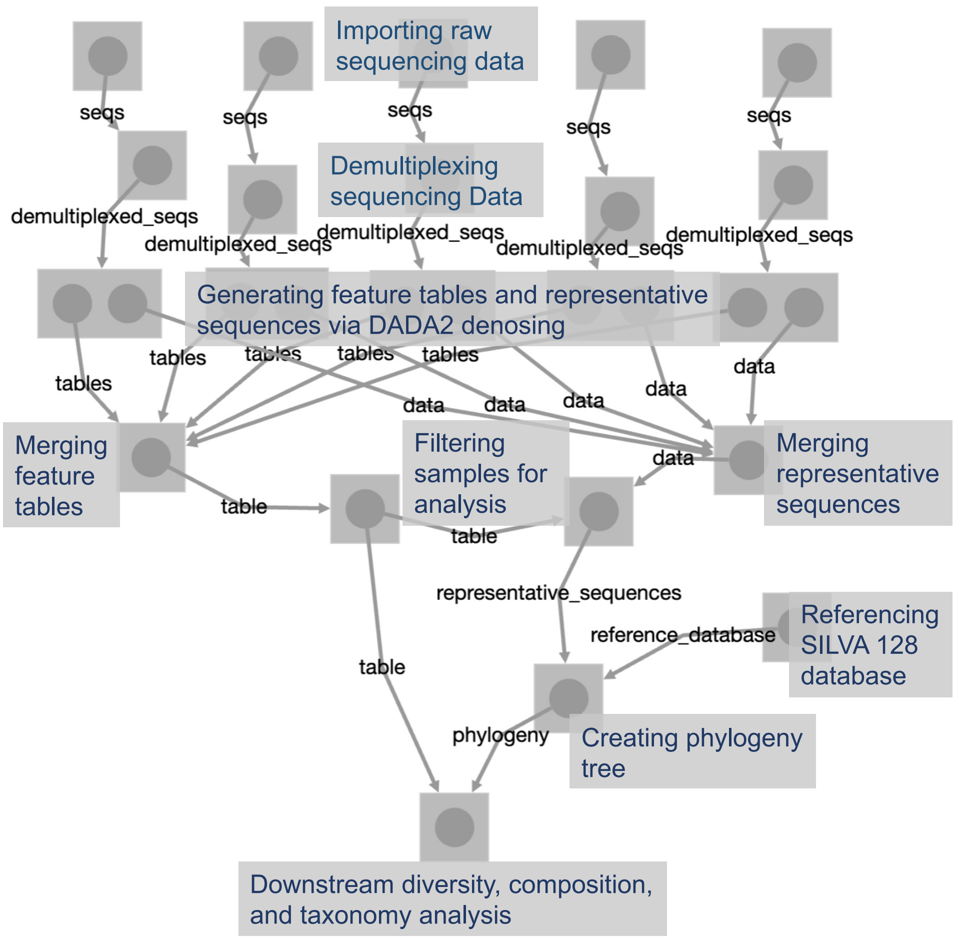


**B** Alpha Rarefaction Curve

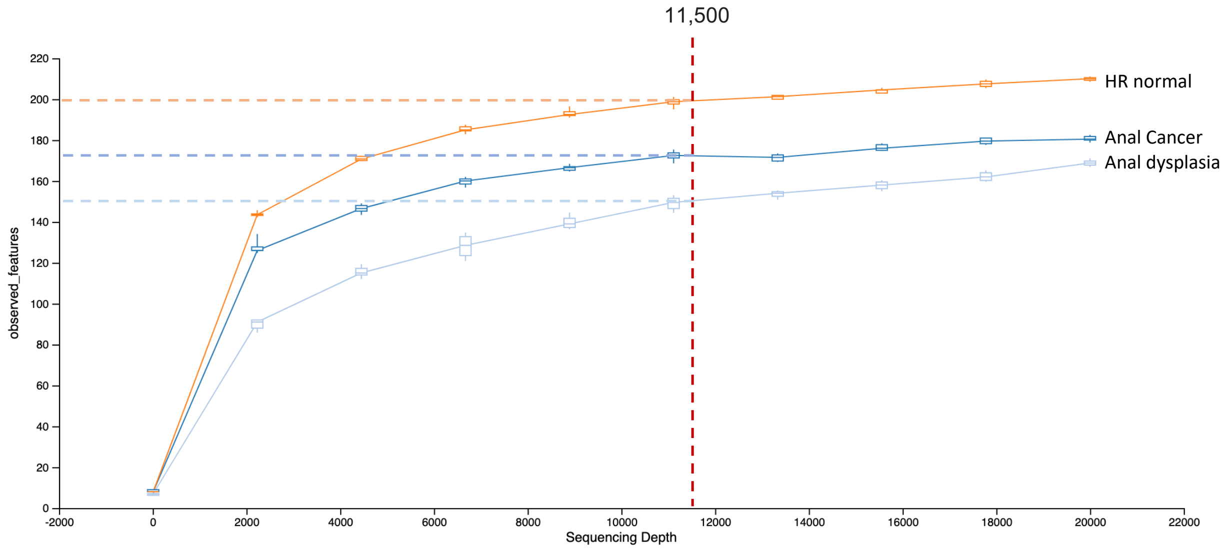

Supplement: Supplementary file 1 [file DataSheet_1.pdf]
