# Supplementary figures and images for: HPV-related anal cancer is associated with changes in the anorectal microbiome during cancer development
Source: Front Immunol. 2023 Mar 29;14:1051431. doi: 10.3389/fimmu.2023.1051431 (PMC10090447; doi:10.3389/fimmu.2023.1051431)

## Supplemental Figure 2 – Alpha diversity

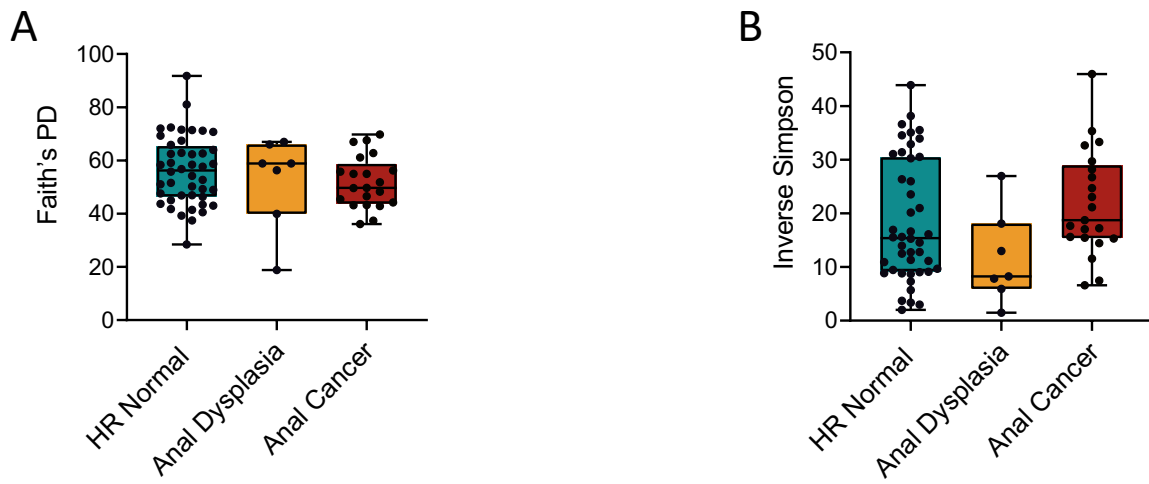

Statistical tests were performed using one way ANOVA. \*P < 0.05

Supplement: Supplementary file 2 [file DataSheet_2.pdf]

### HR Normal vs Anal Cancer Stacked Bar

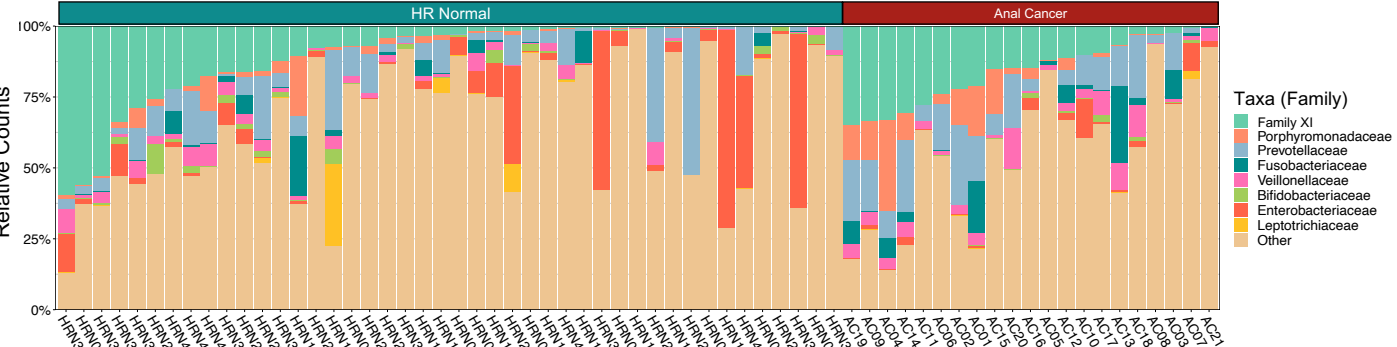

Supplement: Supplementary file 6 [file DataSheet_6.pdf]

# Supplemental Figure 7 – Taxa comparisons

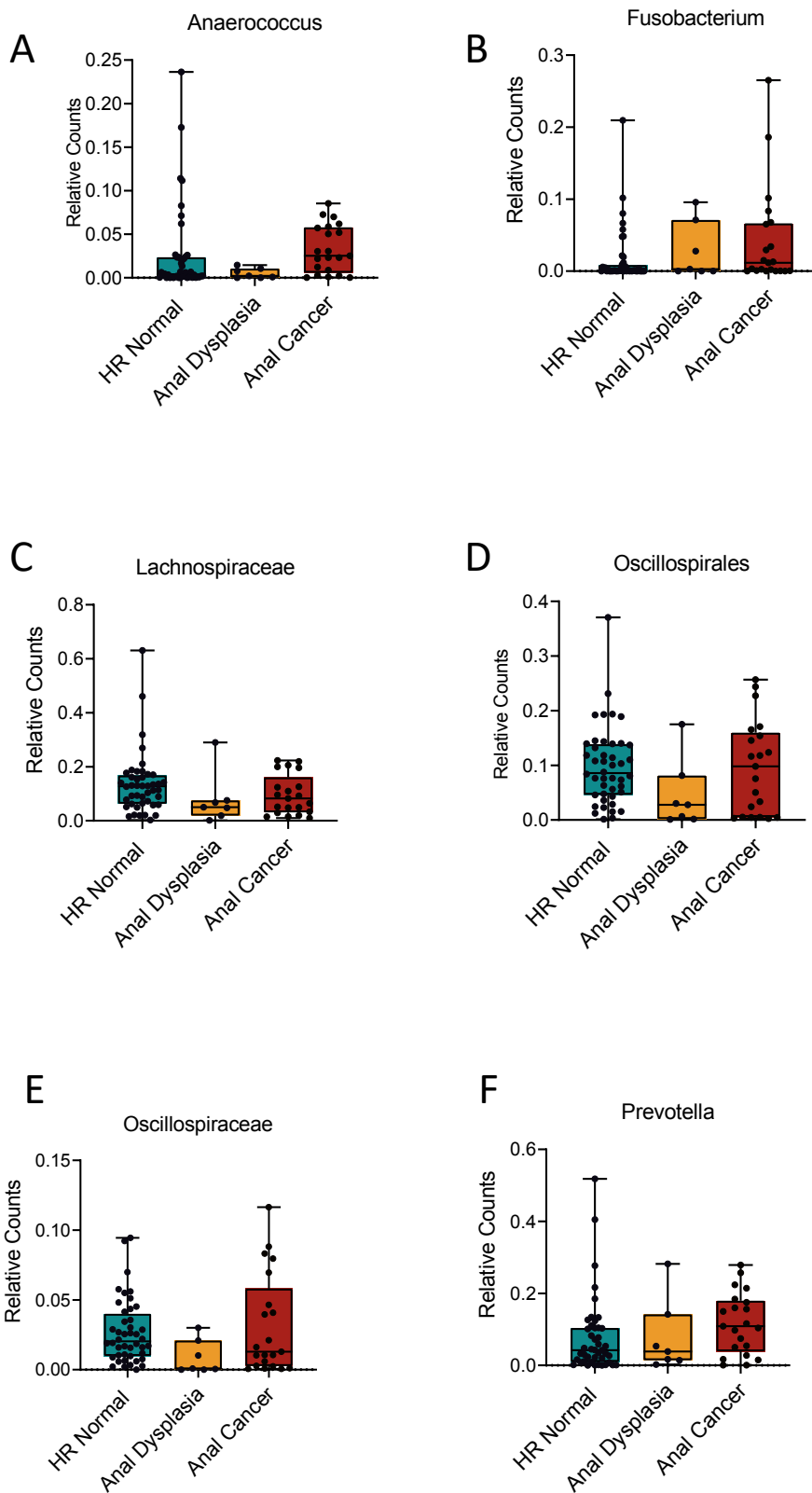

Statistical tests were performed using one way ANOVA. \*P < 0.05

Supplement: Supplementary file 7 [file DataSheet_7.pdf]
