# Supplementary material for: HPV-related anal cancer is associated with changes in the anorectal microbiome during cancer development
Source: Front Immunol. 2023 Mar 29;14:1051431. doi: 10.3389/fimmu.2023.1051431 (PMC10090447; doi:10.3389/fimmu.2023.1051431)

Supplemental Figure 3 – Taxa Relative Counts (Genus)

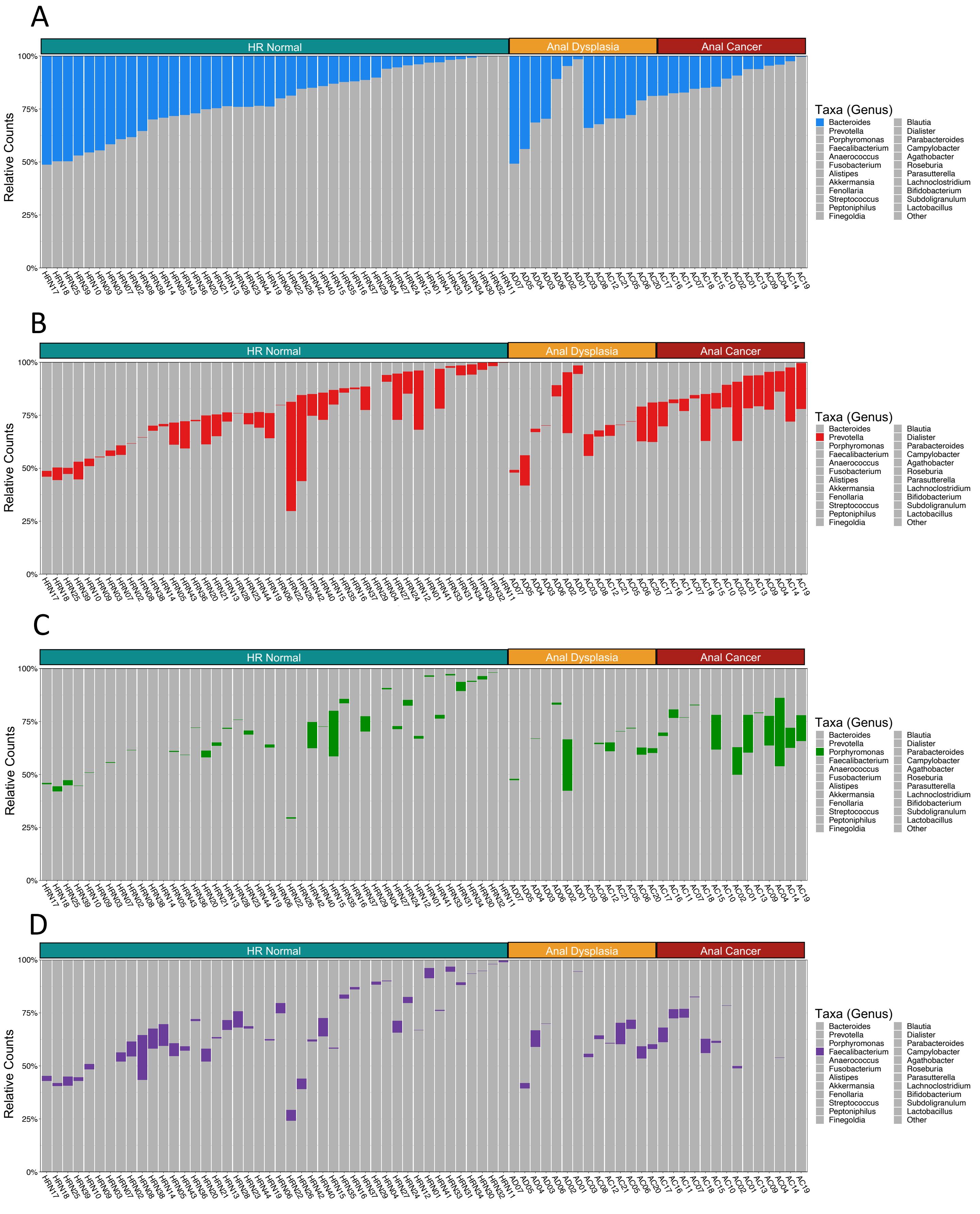

Supplemental Figure 3 – Taxa Relative Counts (Class)

E

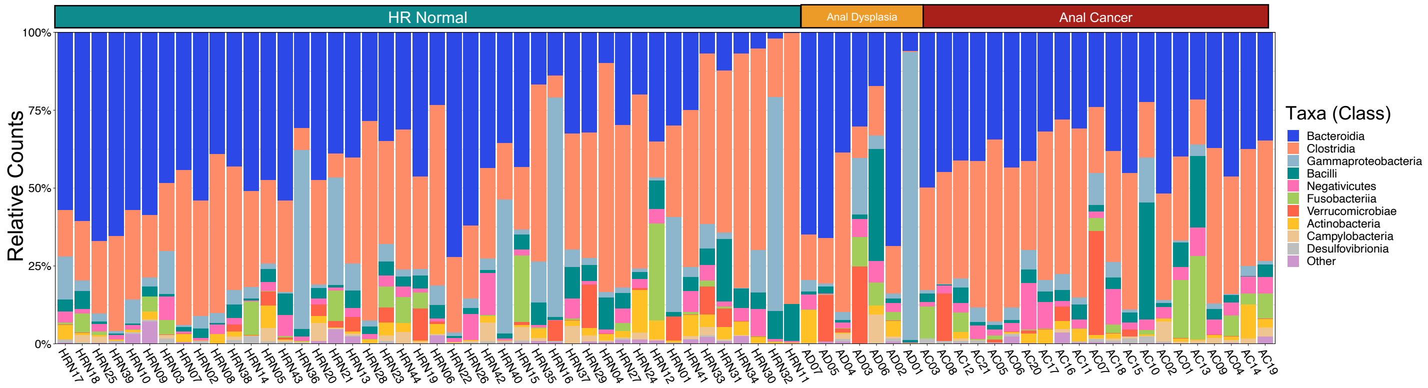

F

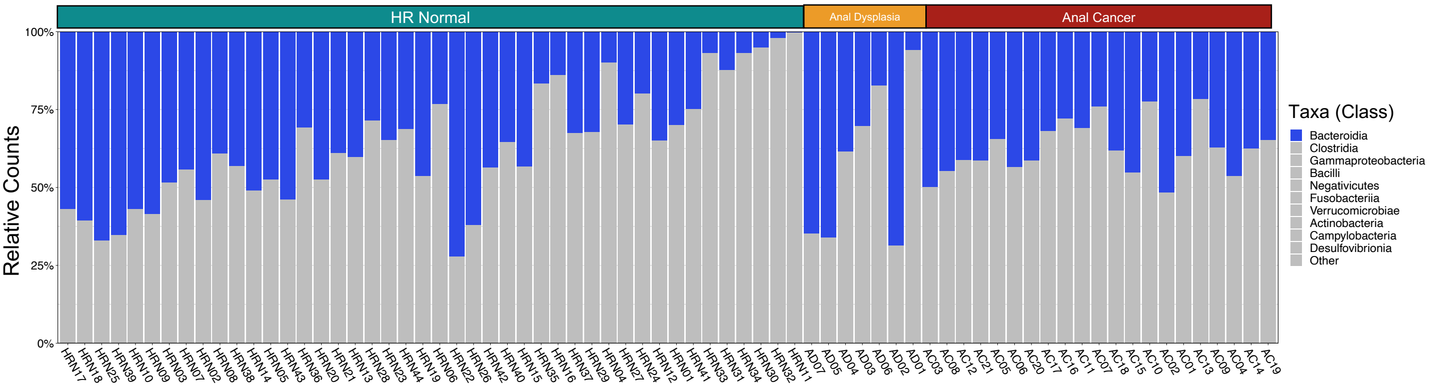

G

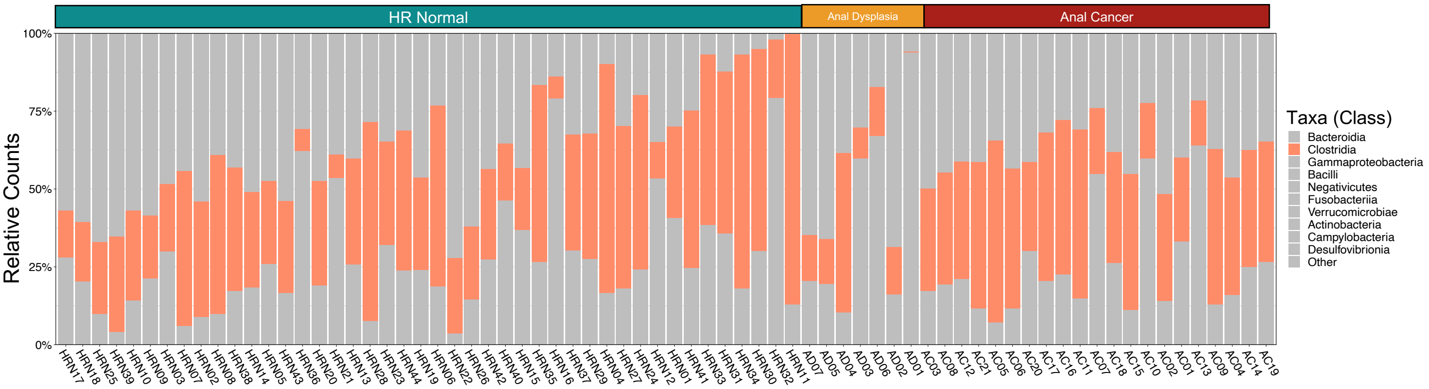

Supplement: Supplementary file 3 [file DataSheet_3.pdf]
