# Supplementary material for: HPV-related anal cancer is associated with changes in the anorectal microbiome during cancer development
Source: Front Immunol. 2023 Mar 29;14:1051431. doi: 10.3389/fimmu.2023.1051431 (PMC10090447; doi:10.3389/fimmu.2023.1051431)

Supplemental Figure 4 – PCoA 3 way

A

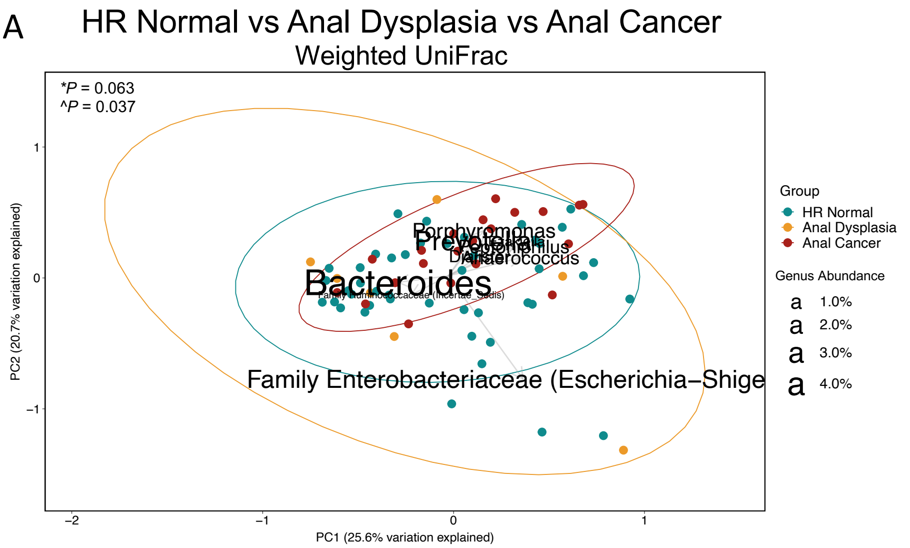

B

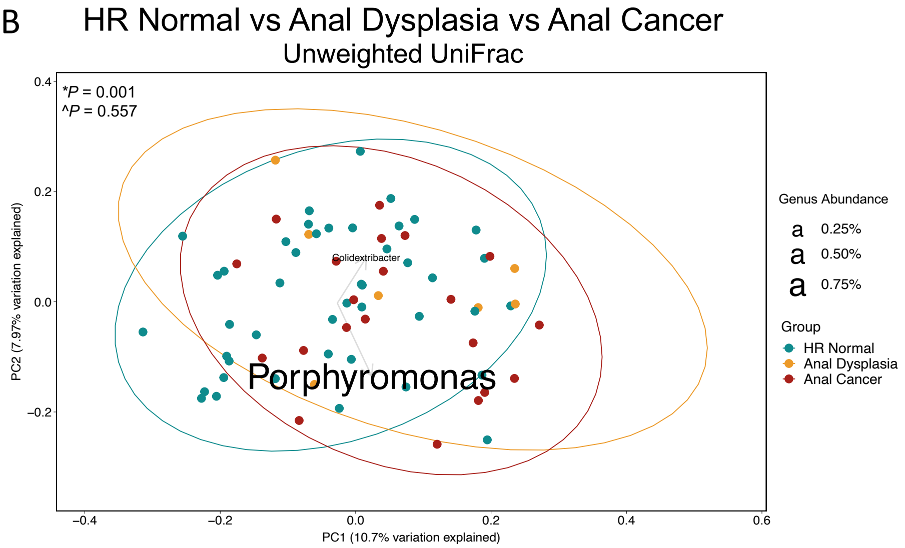

C

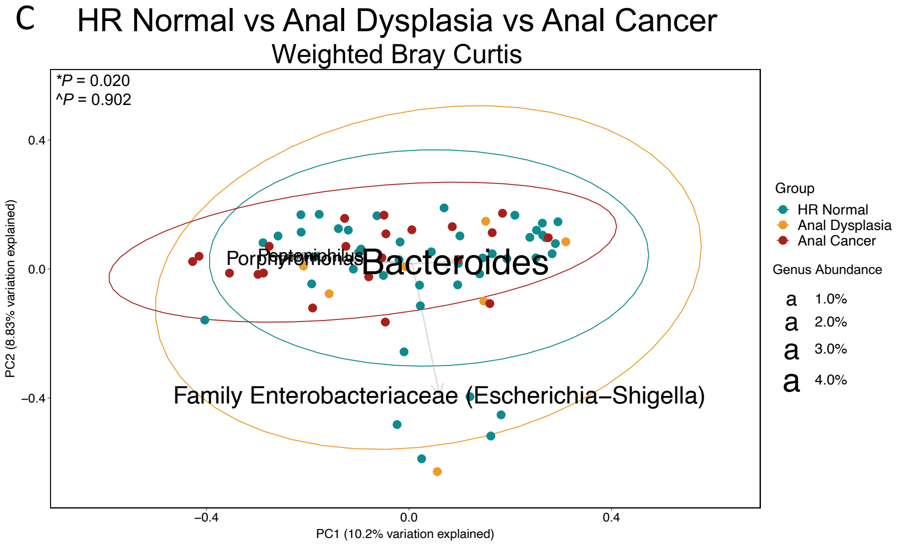

D

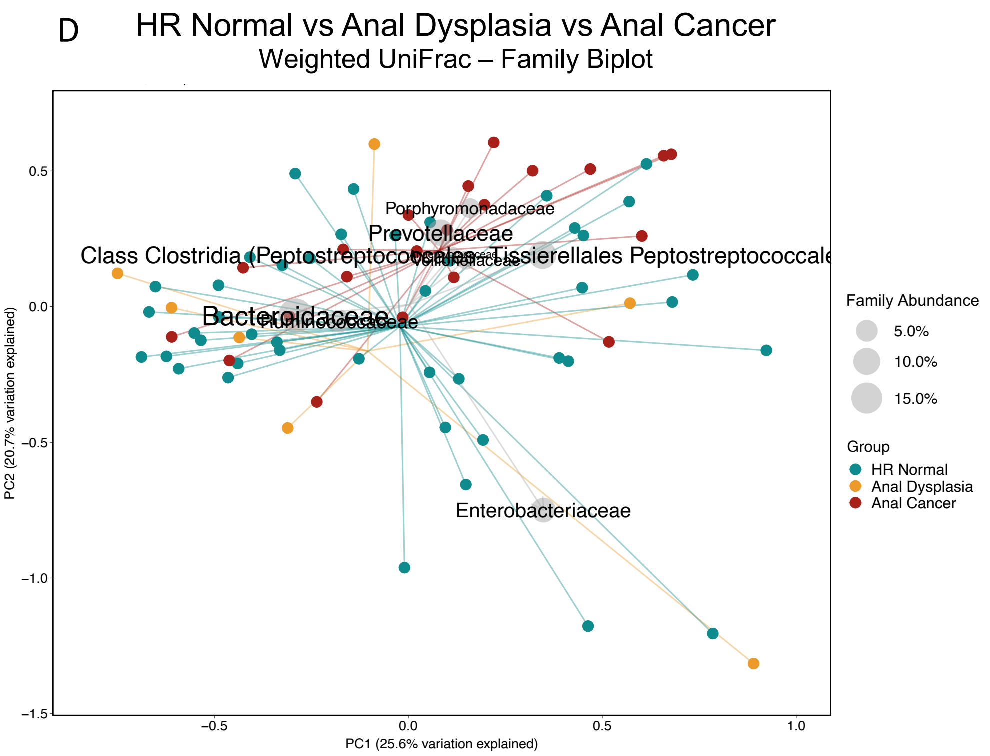
HR Normal vs Anal Dysplasia vs Anal Cancer
Weighted UniFrac – Family Biplot
Porphyromonadaceae
Prevotellaceae
Bacteroidaceae
Enterobacteriaceae
PC1 (25.6% variation explained)
PC2 (20.7% variation explained)
Family Abundance
5.0%
10.0%
15.0%
Group
HR Normal
Anal Dysplasia
Anal Cancer

E

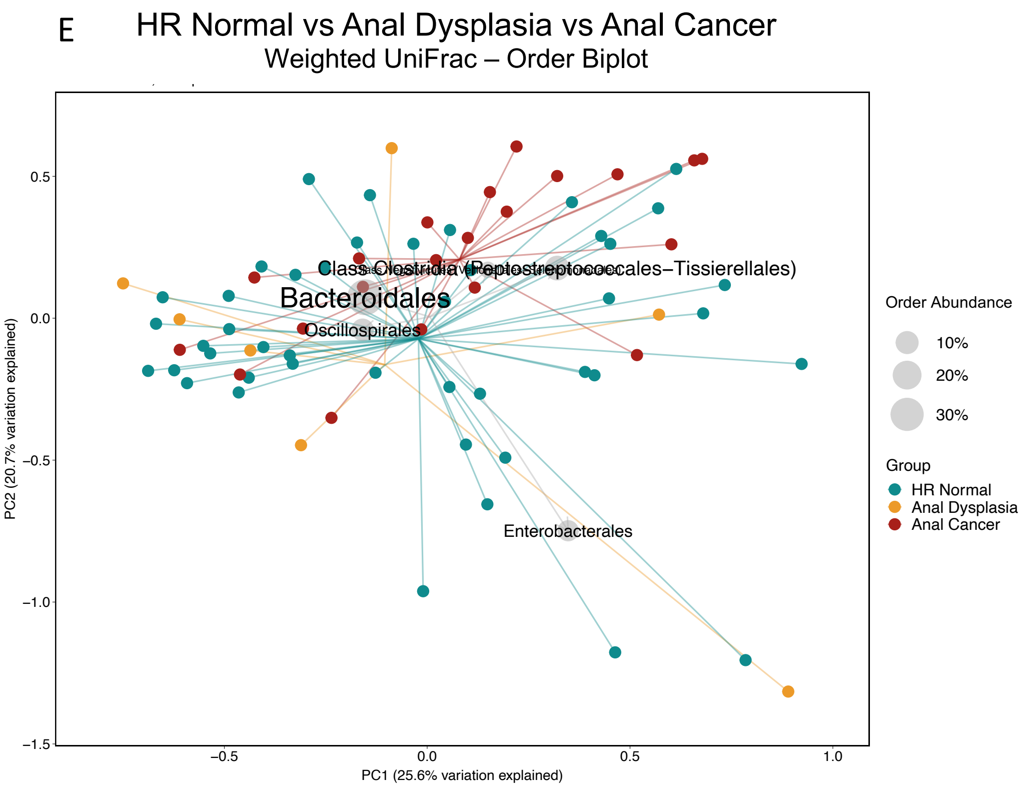
HR Normal vs Anal Dysplasia vs Anal Cancer
Weighted UniFrac – Order Biplot
Bacteroidales
Oscillospirales
Enterobacterales
PC1 (25.6% variation explained)
PC2 (20.7% variation explained)
Order Abundance
10%
20%
30%
Group
HR Normal
Anal Dysplasia
Anal Cancer

Supplemental Figure 4 – PCoAs pairwise UniFrac

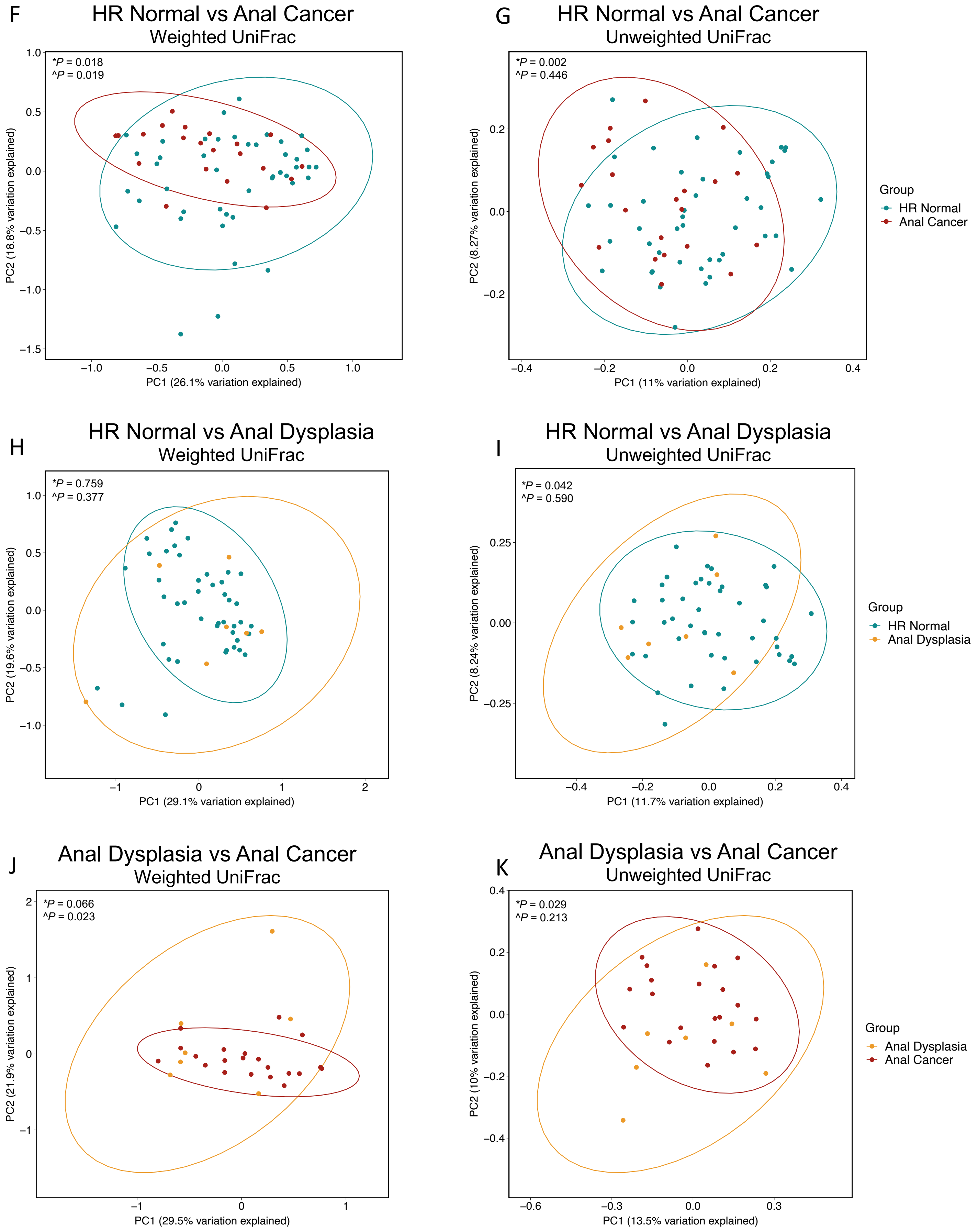

Supplemental Figure 4 – PCoAs Pairwise Bray Curtis

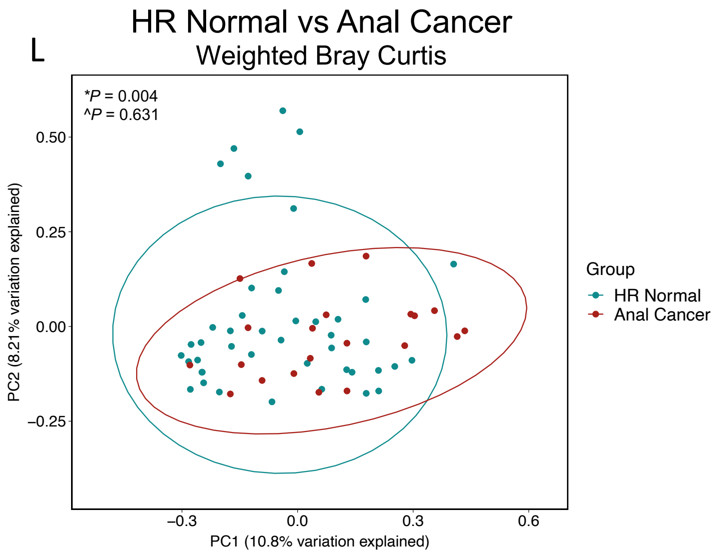

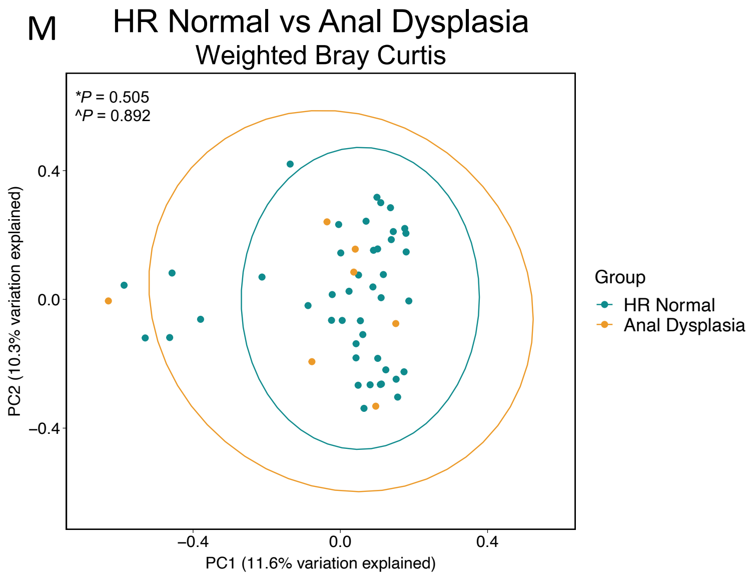

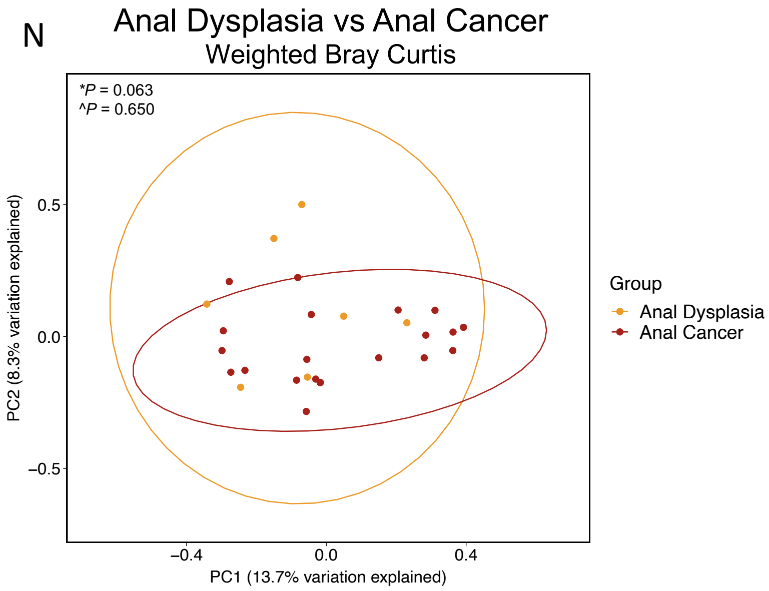

Supplement: Supplementary file 4 [file DataSheet_4.pdf]
