# Supplementary material for: HPV-related anal cancer is associated with changes in the anorectal microbiome during cancer development
Source: Front Immunol. 2023 Mar 29;14:1051431. doi: 10.3389/fimmu.2023.1051431 (PMC10090447; doi:10.3389/fimmu.2023.1051431)

Supplemental Figure 5 – LEfSe

A
HR Normal, Anal Dysplasia, and Anal Cancer Cladogram

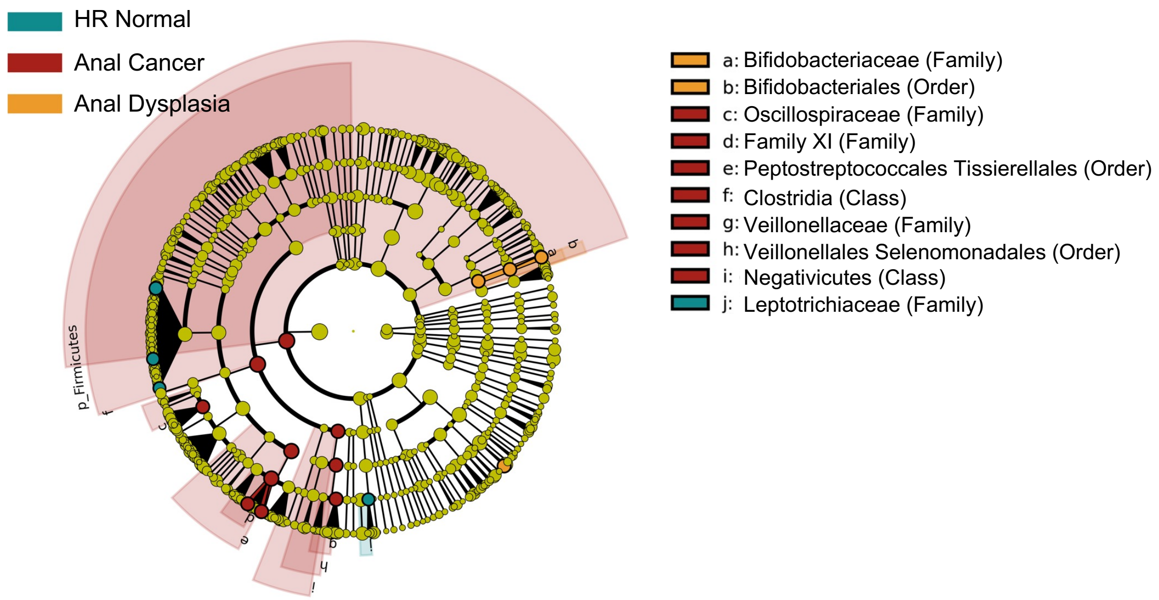

B
HR Normal vs Anal Cancer Cladogram

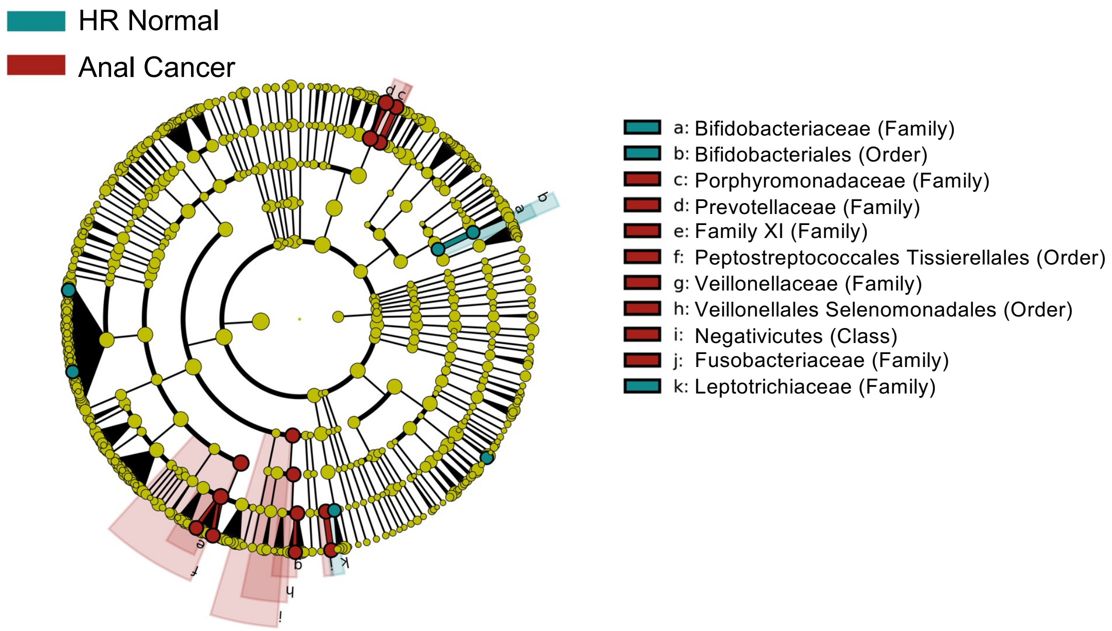

Supplemental Figure 5 – LEfSe

C

HR Normal vs Anal Dysplasia

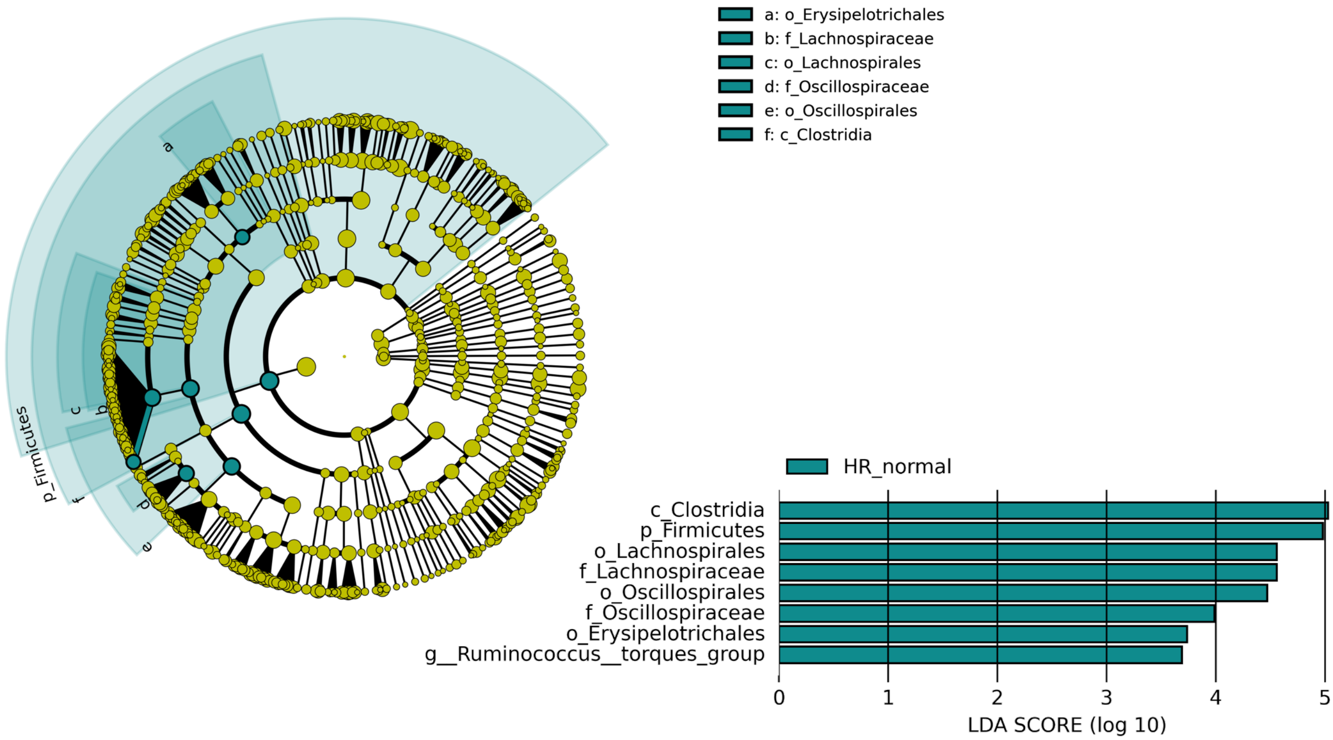

D

Anal Dysplasia vs Anal Cancer

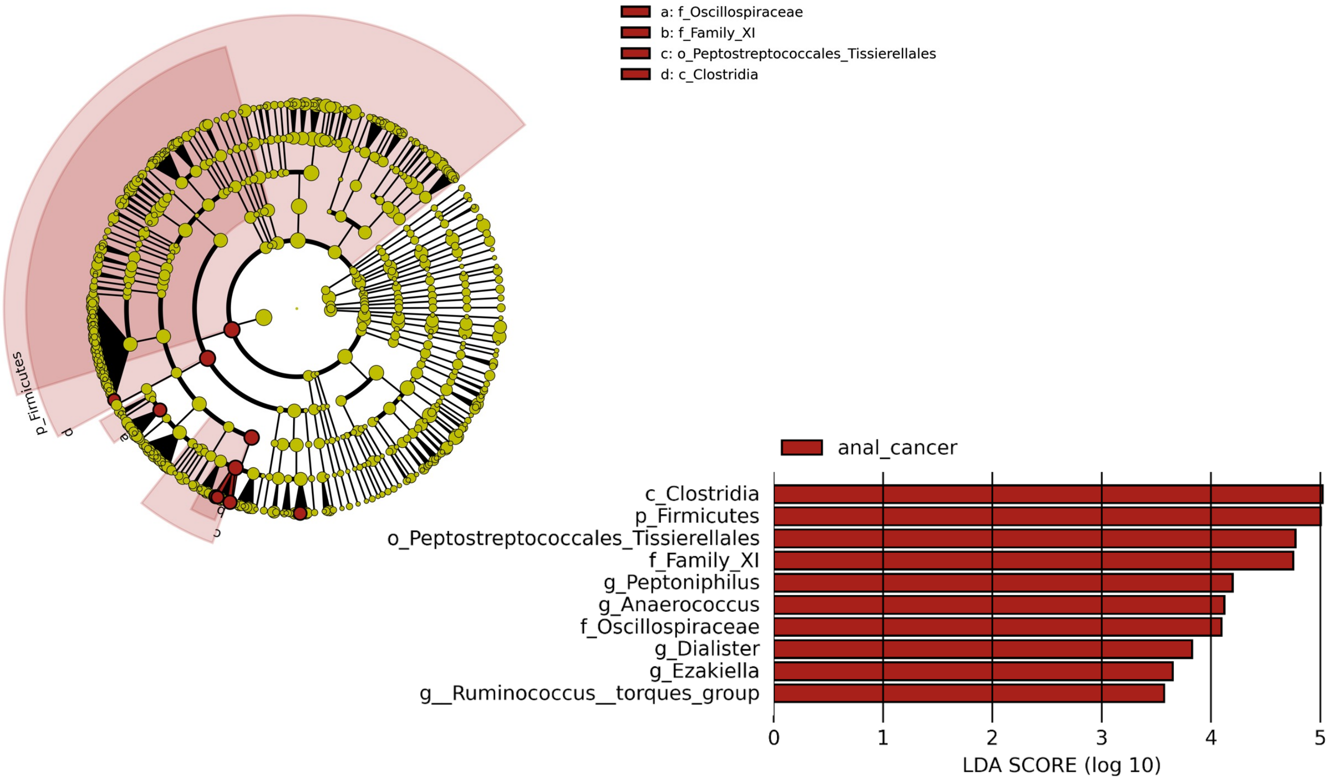

Supplement: Supplementary file 5 [file DataSheet_5.pdf]
